# Microglia at the Tripartite Synapse during Postnatal Development: Implications for Autism Spectrum Disorders and Schizophrenia

**DOI:** 10.3390/cells12242827

**Published:** 2023-12-13

**Authors:** Laura Ferrucci, Iva Cantando, Federica Cordella, Silvia Di Angelantonio, Davide Ragozzino, Paola Bezzi

**Affiliations:** 1 Department of Physiology and Pharmacology, University of Rome Sapienza, 00185 Rome, Italy; laura.ferrucci@uniroma1.it (L.F.); federica.cordella@uniroma1.it (F.C.); silvia.diangelantonio@uniroma1.it (S.D.A.); davide.ragozzino@uniroma1.it (D.R.); 2 Department of Fundamental Neurosciences, University of Lausanne, 1005 Lausanne, Switzerland; iva.cantando@unil.ch; 3 Center for Life Nano- & Neuro-Science, IIT, 00161 Rome, Italy; 4 IRCCS Santa Lucia Foundation, 00179 Rome, Italy

**Keywords:** tripartite synapse, microglia, astrocytes, autism, schizophrenia, synaptic activity, inflammation

## Abstract

Synapses are the fundamental structures of neural circuits that control brain functions and behavioral and cognitive processes. Synapses undergo formation, maturation, and elimination mainly during postnatal development via a complex interplay with neighboring astrocytes and microglia that, by shaping neural connectivity, may have a crucial role in the strengthening and weakening of synaptic functions, that is, the functional plasticity of synapses. Indeed, an increasing number of studies have unveiled the roles of microglia and astrocytes in synapse formation, maturation, and elimination as well as in regulating synaptic function. Over the past 15 years, the mechanisms underlying the microglia- and astrocytes-dependent regulation of synaptic plasticity have been thoroughly studied, and researchers have reported that the disruption of these glial cells in early postnatal development may underlie the cause of synaptic dysfunction that leads to neurodevelopmental disorders such as autism spectrum disorder (ASD) and schizophrenia.

## 1. Introduction

Neurodevelopmental disorders constitute a diverse spectrum of conditions with complex manifestations, impacting motor, sensory, and cognitive functions from early childhood through adolescence and into adulthood [1,2]. Within this broad classification, autism spectrum disorders (ASDs) and schizophrenia (SZ) have emerged as significant focal points, presenting distinct neurological and psychiatric features that contribute to a substantial disease burden worldwide [3,4]. ASDs, typically identifiable in early childhood, manifest through persistent deficits in social communication, repetitive behaviors, and restricted interests, with a prevalence of 1 in 100 and a fourfold predominance in males [5,6]. SZ, affecting around 1% of the population, primarily surfaces during late adolescence and early adulthood and is characterized by positive, negative, and cognitive symptoms [7,8].

While the etiology of these disorders remains multifaceted, emerging evidence highlights the intricate involvement of the immune system, particularly microglial cells, in the pathophysiology of ASDs and SZ [9,10]. Perturbations in circulating cytokine profiles, alterations in inflammatory markers, and genetic associations within the major histocompatibility complex (MHC) region underscore the critical role of the immune response in these conditions [11,12]. Environmental factors, including prenatal infections and maternal immune activation, have been implicated in contributing to the risk of developing these disorders, further emphasizing the intricate interplay between genetic predisposition and environmental influences [13,14].

Notably, the significance of glial cells, particularly microglia, and astrocytes in orchestrating neurodevelopmental trajectories and synaptic plasticity has garnered substantial attention in the last decade. Microglia, integral components of the brain’s innate immunity, are intricately involved in the pathology of neurodevelopmental disorders, while astrocytes actively modulate synaptic activity and exert regulatory functions within the central nervous system. Considering the intricate relationships between glial cells and neuronal elements in the context of physiological and pathological conditions, especially those related to ASD and SZ, the exploration of the tripartite synapse [15,16] assumes critical importance. Integrating the findings from human-based research and technological advances provides a comprehensive understanding of the molecular and cellular mechanisms underlying the complex interplay between glial cells and synaptic activity in the context of these neurodevelopmental disorders. By unraveling the intricate contributions of glial cells, we can pave the way for targeted therapeutic interventions that aim to restore the delicate balance between neuroimmune responses and synaptic function, thereby offering promising avenues for the treatment of ASDs and SZ.

## 2. Microglia Sense the Tripartite Synapse during Postnatal Development

**Figure 1:**
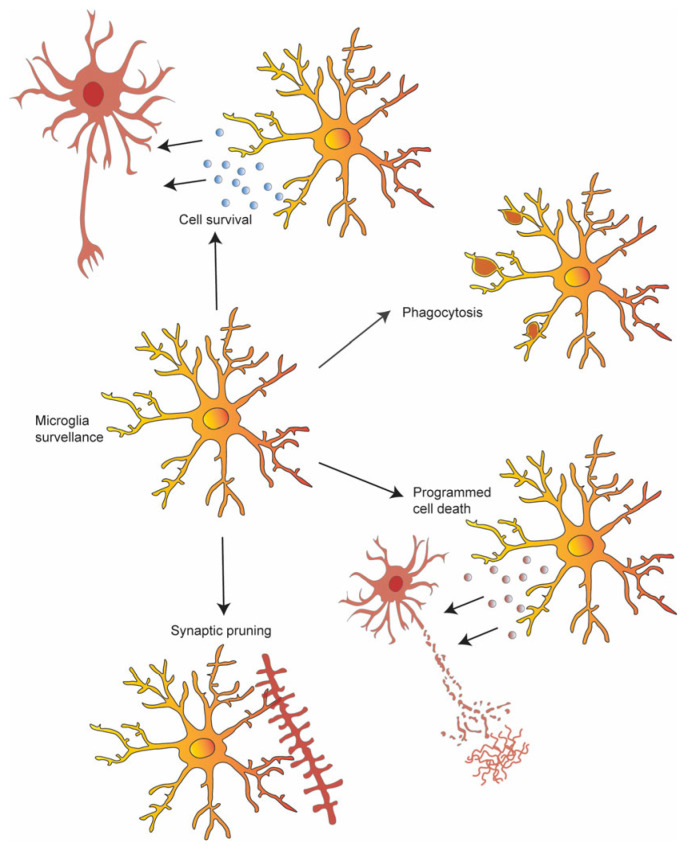
Overview of the main functions carried out by microglia in the healthy brain. In the healthy brain, microglia move their processes to monitor the surrounding microenvironment, promote neuronal survival, or participate in neuronal cell death, even phagocytosing cell debris. Moreover, microglia shape neuronal circuits by actively phagocytosing supernumerary synapses.

Microglia, highly dynamic cells, continuously survey their microenvironment by extending and retracting fine processes [17,18], performing key functions in the healthy brain (Figure 1). Several investigations using in vivo imaging and high-resolution electron microscopy (EM) in the healthy mouse cortex during postnatal development have revealed that the fine processes of microglia actively form associations with synapses [19,20,21,22,23,24]. This suggests that these cells can sense neuronal activity and actively remodel synaptic circuits.

Microglia are equipped with a wide array of classical neurotransmitter receptors, including those for purines, γ-aminobutyric acid (GABA), dopamine, and glutamate [25,26]. The majority of their receptors are G-protein-coupled receptors (GPCRs) coupled with an increase in cytosolic calcium (Ca^2+^) [27,28]. Several neurotransmitter receptors, such as GABAB [29,30], histamine, dopamine [31], serotonin [32], and muscarinic acetylcholine [33], have been functionally identified in microglia through live-cell Ca^2+^ imaging techniques using freshly isolated microglia or primary cultured cells. Recent findings utilizing Ca^2+^ imaging techniques and multiple genetically encoded calcium indicator GCaMP6 variants targeted to microglia assessed how microglial Ca^2+^ signaling responds to alterations in neuronal activity. These findings showed that microglia have highly distinct microdomain signaling and that processes specifically respond to bi-directional shifts in neuronal activity (i.e., increase or decrease) through increased calcium signaling [34,35]. These data raise the possibility that endogenous neurotransmitter receptors on microglia enable them to detect changes in synaptic activity [36]. Indeed, a recent study highlighted that microglia sense neuronal synaptic activity through a signaling pathway involving astrocytic GABA and glutamate transporters, as well as microglial GABAB receptors [37]. The identity and location of glutamate and GABA transporters were demonstrated by the use of a potent and selective glutamate transporter inhibitor TFB-TBOA, which specifically blocks excitatory amino acid 1 and 2 transporters (EAAT1/2) on astrocytes but not neuronal EAAT3 [38,39,40]. Astrocytic glutamate transporters EAAT1/2 and GABA transporters uptake glutamate and GABA released from presynaptic terminals, utilizing a Na^+^ gradient as an energy source, leading to intracellular Na^+^ increases [41]. The increase in intracellular Na^+^ levels triggers GABA release through reversed GABA transport in astrocytes [42], which is then detected by microglial GABAB receptors. This study observed an average delay of 3.4 s in microglial Ca^2+^ responses compared to astrocytic stimulation, consistent with GABA transporter type 3 (GAT3) reversal [43]. Furthermore, microglial response rates to external GABA application exceeded those of electrical stimulation, suggesting that bath-applied GABA can activate all microglial GABA receptors [42]. Importantly, microglial responses to neuronal stimulation were only observed during early postnatal stages and were absent in adulthood, suggesting that the GABA receptors in developing microglia may be important for the postnatal maturation of neuronal circuits. Confirming this hypothesis, a recent study showed that microglia play a direct role in interacting with inhibitory synapses and that this interaction is notably weakened in GABAB1 receptor conditional knockout mice (cKOs) [30]. In this study, the authors showed that the microglia expressing GABAB1 receptors preferentially establish contacts with inhibitory synapses over excitatory ones and that these connections decrease when GABAB receptors are removed from microglia, thus indicating that a subgroup of microglial cells is targeted specifically to inhibitory synapses. Transcriptomic analyses conducted independently confirm that the elimination of microglial GABAB receptors results in selective changes in the synapse-pruning genes within GABA-receptive microglia. Importantly, the removal of GABAB receptors from microglia affects their inhibitory connectivity while leaving excitatory synapses unaffected. These cKOs also exhibit behavioral abnormalities that align with the alterations observed in inhibitory synaptic function.

Microglia exhibit an extraordinary sensitivity to neuronal activity, spanning from the level of single neurons to broader circuit excitability (as comprehensively reviewed in [44]). In the intact brain, microglial processes frequently engage spontaneously active neurons, including those responsive to visually evoked stimuli [45,46]. Notably, even a single neuron firing a train of action potentials in acute brain slices can evoke responses from nearby microglial processes [47], highlighting the remarkable selectivity of microglial responses to single active neurons within a field of many. This principle extends to brain circuits; for instance, high-frequency stimulation of the Schaffer collateral pathway in acute hippocampal brain slices leads to denser microglial sub-process outgrowth and prolonged contact time with hippocampal neurons [48]. The role of neuronal activity in engaging microglial process responses has also been demonstrated using pharmacological manipulations such as the removal of extracellular calcium or glutamate application, known to enhance neuronal activity [49,50]. In in vivo experiments, chemogenetic approaches utilizing Gq DREADDs to activate a small neuronal population lead to an increased microglial process area and enhanced process contact with neuronal somata [34,51]. Similarly, a topical application of bicuculline, a GABA receptor antagonist, heightened microglial territory surveillance [18].

Not only microglia but also astrocytes detect the spillover of neurotransmitters released during synaptic transmission through a wide range of neurotransmitter receptors and transporters [52]. In response to neuronal activity, astrocytes release gliotransmitters [53,54], such as glutamate, adenosine triphosphate (ATP), adenosine, d-serine, tumor necrosis factor alpha (TNFα), and GABA [52,53,54,55,56,57,58,59,60,61,62,63,64,65], thereby providing feedback to the neuronal network. An intriguing possibility is that astrocyte-released gliotransmitters may also act on receptors located on microglia. For example, purinergic signaling, facilitated by the P2Y receptors found on both astrocytes and microglia, likely plays a crucial role in mediating communication between these two cell types and synaptic activity. Regarding the mechanisms implicated in the neuron–microglia communication system, purinergic signaling represents the most extensively studied and characterized mechanism involved in microglial responses to synaptic activity. During heightened neuronal activity, the release of ATP and adenosine diphosphate (ADP) from neurons acts as a signal to activate the microglial receptor P2Y12. This activation represents a well-preserved response mechanism not only triggered in response to localized tissue damage as initially demonstrated [18] but also employed in situations of excessive neural excitability.

Purinergic communication involves astrocytes as well. An illustrative example of this interplay involves the ATP released from synaptic activity at the perforant path (PP)-granule cell (GC) synapses in the hippocampal dentate gyrus [66]. ATP is able to trigger intracellular Ca^2+^ elevations in nearby astrocytes of the dentate molecular layer (ML) by activating purinergic P2Y1 receptors (P2Y1R), which in the ML are expressed predominantly, if not exclusively, in astrocytes, most notably in the processes surrounding excitatory synapses [66]. Because of P2Y1R-dependent intracellular Ca^2+^ elevation, glutamate is released from astrocytes via a mechanism that is sensitive to the blockers of neuronal exocytosis [67] and induces potentiation of excitatory transmission at PP-GC synapses. This glial glutamatergic control operates under physiological conditions on presynaptic NMDA, particularly on NR2B subunits that generally face perisynaptic astrocytic processes containing groups of small vesicular organelles (synaptic-like microvesicles) [57]. Interestingly, the P2Y1-mediated glutamate released by astrocytes is under the control of the cytokine TNFα [67,68]. TNFα is mostly regarded as a proinflammatory cytokine, produced in the brain in response to infection, injury, or disease [56]. However, TNFα is also expressed in the normal brain, albeit at much lower levels than during inflammatory reactions, and participates in homeostatic brain functions such as the stability of neuronal networks in response to prolonged changes in activity via the phenomenon of synaptic scaling [69,70]. This cytokine, released from astrocytes, was reported to strengthen excitatory synaptic transmission by promoting the surface insertion of AMPA receptor subunits [71,72] and to exert an obligatory factor for the induction of synaptically effective gliotransmission at GC synapses in the dentate gyrus, specifically controlling glutamate release from astrocytes [68].

Several studies have shown that microglial cells can change their motility in response to ATP or other purinergic signaling released by injured cells in response to tissue damage [17,18,73,74,75,76]. The ATP or other purinergic signaling can also be released by astrocytes [77] and can bind to specific P2Y receptors, namely P2Y12 and P2Y6, present on microglia, prompting microglial responses, such as phagocytosis and the extension of cellular processes [78], or the production of inflammatory cytokines by microglia [79]. This intricate purinergic signaling network demonstrates how the release and reception of ATP, mediated by P2Y receptors in both microglia and astrocytes, contribute to the regulation of immune responses and intercellular communication. While the specific role of gliotransmitters in mediating astrocyte–microglia interactions requires further investigation, it is plausible that the gliotransmitters released by astrocytes could serve as signaling molecules that inform the microglia about changes in synaptic activity and guide their responses, such as phagocytosis of synaptic elements.

## 3. Microglia and Astrocytes Sculpt the Tripartite Synapse during Postnatal Development

Microglia play a critical role in regulating neural circuit development in the brain [77,80], with extensive evidence highlighting their influence on various aspects, including triggering neuronal apoptosis and governing the process of synapse formation and elimination. These studies underscore the indispensable involvement of microglia in shaping the development of neural circuits, particularly emphasizing their crucial role at synapses [81,82].

During neurodevelopment, an initial overproduction of cells is followed by a pruning process. Disruptions in the correct number of neurons or synapses can significantly impair normal circuit functioning. In healthy neurodevelopment, programmed cell death, primarily through apoptosis, accounts for most of the natural cell die-off. Microglia have the capability to induce cell death directly through the secretion of noxious factors, such as reactive oxygen species (ROS) and nerve growth factor (NGF) [83,84]. Additionally, they can promote neuron survival by secreting trophic factors and cytokines [85,86]. Studies involving the pharmacological inactivation of microglia with minocycline during early postnatal development have shown an increase in cortical neuron cell death [86]. This observation was further substantiated by subsequent reports of widespread cell death associated with minocycline treatment [87,88]. Notably, this process involved the C-X3-C motif chemokine receptor 1 (CX3CR1)-regulated release of insulin-like growth factor 1 (IGF-1) by microglia [86]. Thus, microglia display a dual role, not only regulating neuronal death but also facilitating neuronal survival during development.

Microglia are actively involved in clearing cellular debris, including whole neuronal cells, during development. They have emerged as crucial players in maintaining the delicate balance of synapse numbers, involved in both elimination and formation. Notably, microglia’s role in shaping synaptic connectivity originates from studies implicating their involvement in the engulfment of synaptic material in the mouse hippocampus, a process dependent on the presence of CX3CR1 [89]. The mechanism underlying microglia-mediated synaptic elimination seems to depend on the interaction between the phagocytic complement receptor on the microglial cell surface and its corresponding ligand, C3 [24,90,91]. This process finds support in the involvement of the classical complement cascade in the developmental elimination of synapses within the central nervous system [92]. The involvement of the C3 complement extends to microglia–astrocyte interactions, where microglia play a vital role in orchestrating the development of the spatially organized astrocyte template necessary for subsequent vascular growth during neuroretina development, acting through the complement C3/C3aR axis [93]. A deficiency in C3 or C3aR disrupts the developmental phagocytic elimination of astrocyte bodies, leading to a noticeable increase in astrocyte density. Subsequent studies in the lateral geniculate nucleus of the visual system have revealed a complement-dependent mechanism in microglia’s regulation of synapse numbers [90,92]. Moreover, microglia participate in controlling synapse elimination in the developing brain, with significant contributions from triggering myeloid cells 2 (TREM2)-mediated signaling, particularly in the hippocampus [94]. The mechanisms governing synapse elimination involve both “eat me” signals, such as phosphatidylserine, and “don’t eat me” signals, like CD47 [95]. Impaired synapse elimination resulting from sensory deprivation was observed in adolescent mice lacking the microglial-specific purinergic receptor P2RY12 [96]. Notably, the microglial regulation of synapse elimination is subject to modulation by interactions with astrocyte-derived factors, such as IL-33 [97].

In addition to their role in synaptic pruning and formation, recent evidence highlights the involvement of microglia in controlling astrocyte numbers in the developing mouse retina, a process reminiscent of phagoptosis [98]. A significant phenomenon observed in retinal development is the substantial reduction in the total number of retinal astrocytes, which decreases by more than threefold during a specific period from postnatal days 5 to 14. Interestingly, this decline in astrocyte numbers does not occur through apoptosis, the conventional process responsible for most developmental cell deaths. Instead, this study revealed a different mechanism at play: microglia actively participate in eliminating astrocytes during this developmental phase. When microglia were genetically removed from the equation, a significant inhibition of astrocyte death was observed, resulting in a larger population of astrocytes persisting at the conclusion of the death period. However, even in the absence of microglia, astrocyte death was not completely halted, attributed to the ability of astrocytes to engulf one another. Nevertheless, mice lacking microglia exhibited substantial structural changes in their retinal astrocyte network, ultimately leading to the occurrence of retinal hemorrhages.

Notably, not only microglia but also astrocytes have a role in synaptic pruning. Synaptic phagocytic receptors MEGF10 and MerTK are expressed on the surface of astrocytes. Astrocytes can eliminate excitatory or inhibitory synapses by interacting with MEGF10 and MerTK, which can recognize phosphatidylserine in target fragments as an opsonic signal to initiate phagocytosis and drive synaptic remodeling [99]. This process of synaptic elimination is strongly dependent on neural activity. Recent studies have shown that astrocytes regulate synaptic elimination by activating purinergic signals in an ATP-dependent manner mediated by inositol 1,4,5-trisphosphate (IP3) receptor (IP3R) type 2 (IP3R2) release [100]. Additionally, astrocytes can express Ephrin-B1, which is a membrane-binding protein and the ligand of the EphB receptor. The cross-synaptic ephrin-B/EphB interaction between neurons is essential for the formation and maintenance of synapses in the mouse brain [101,102,103]. Astrocytic ephrin-B1 can compete with neuronal ephrin-B1 and trigger the astrocyte-mediated elimination of synapses containing EphB receptors through cross-synaptic endocytosis. The loss of the neuronal EphB receptor weakens the ability of astrocytes expressing functional Ephrin-B1 to phagocytize synaptosomes in vitro, while the overexpression of Ephrin-B1 in astrocytes impairs long-term contextual memory [104].

Overall, these findings shed light on the intriguing interplay between microglia and astrocytes in orchestrating retinal development during this critical period. Moreover, since astrocytes are critical regulators of synapse and circuit development [105,106,107,108], microglial control over astrocytes indirectly influences the orchestration of normal circuit development.

## 4. Microglia at the Tripartite Synapse in ASD and Schizophrenia

**Figure 2:**
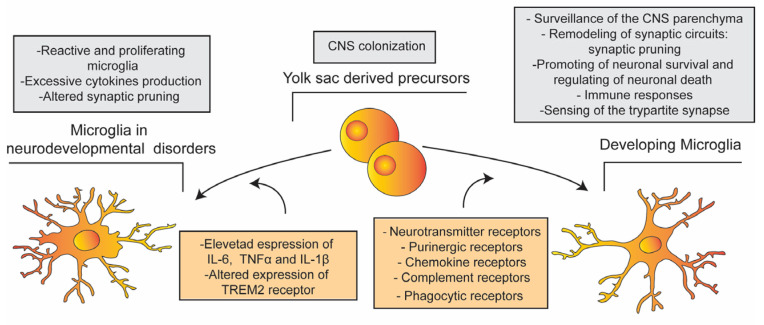
Microglia function in the healthy brain and neurodevelopmental disorders. Schematic summary of the development of microglia and their functional alterations observed in neurodevelopmental disorders.

Emerging evidence has shed light on dysfunctions in microglia and astrocytes in neuro-developmental disorders such as ASD and SZ, suggesting their potential involvement in the initiation of behavioral and cognitive deficits associated with these pathologies [107,109,110,111,112,113] (Figure 2). The significance of microglia and astrocytes in the pathophysiology of neurodevelopmental disorders is predominantly supported by recent transcriptomic analyses across psychiatric conditions. For instance, RNA sequencing has revealed a close association between ASD and SZ, with genes linked to glial cell activation, immunity, and inflammation [114,115,116], as well as an enrichment in microglia and pruning-related modules in ASD patients [115]. Similarly, extensive genome-wide association studies have identified several causal genes related to SZ, including ATP2A2, PSMA4, PBRM1, SERPING1, and VRK2, all highly expressed in microglia [117], as well as genes associated with astrocyte specification and maturation, such as SOX9, GJA1, SPON1, and NOTCH2 [115].

These meta-analyses have been reinforced by evidence derived from the postmortem brain tissues of autistic and schizophrenic patients which demonstrated an activated state of microglial cells characterized by amoeboid morphology, excessive cytokine production, and cell proliferation [118,119,120,121,122,123,124,125,126,127,128,129]. Additionally, significant changes in astrocyte density, morphology, and the deregulated expression of common astrocyte markers like glial fibrillary acidic proteins (GFAP), aquaporin 4 (AQ-4), S100β, glutaminase, thrombospondin (TSB-1), and EAAT2 have been reported [130,131,132,133]. Notably, recent positron emission tomography (PET) functional imaging studies have revealed microglial activation in various brain regions of young adults with ASD and SZ [125,134]. Bloomfield et al. (2016) indicated a significantly higher binding ratio of the second-generation translocator protein (TSPO) radioligand [11C] peripheral benzodiazepine receptor 28 (PBR28), reflecting the number of activated microglia with brain positron emission tomography (PET), in patients with SZ and individuals at ultra-high risk of psychosis, compared to healthy controls. Moreover, this ratio positively correlated with the severity of symptoms in SZ patients [135].

In alignment with the hypothesis of microglial activation in patients with autism and SZ, elevated levels of pro-inflammatory cytokines such as interleukin (IL)-6, TNFα, and IL-1β have been documented in the postmortem brain tissues and blood samples of autistic and schizophrenic patients, including those with 22q11 deletion syndrome [119,136,137,138,139,140,141]. Considering these findings, alongside previous postmortem brain imaging and histological evidence, and subject to limitations and inconsistencies, it is plausible to infer that excessive microglial and astrocyte activation occurs in a subset of patients with SZ and ASD.

Recent research utilizing animal models has underscored the critical involvement of both astrocytes and microglial cells in the pathogenesis of ASDs and SZ. Dysfunction of these cells during neurodevelopment leads to a spectrum of cognitive and behavioral abnormalities, including behaviors resembling autism-related obsessive-compulsive traits and social difficulties. Investigations employing specific techniques targeting astrocytes in the prefrontal cortex (PFC) have provided valuable insights into the functional implications of these cells in neurocognitive processes. For instance, the selective elimination of astrocytes in the PFC through the application of an astrocyte-specific toxin L-α-aminoadipate (L-AA) [142] or the use of a transgenic mouse model with inducible and selective tetanus neurotoxin (TeNT) expression in astrocytes [143] has resulted in notable cognitive deficits across various domains. These deficits encompass impaired attentional set-shifting, compromised working memory, and difficulties in reversal learning [142], along with disruptions in recognition memory and abnormal cortical gamma oscillations [143]. Notably, recent studies have implicated the role of astrocytes in the regulation of extracellular dopamine homeostasis in the developing PFC, influencing the onset of repetitive behavior and cognitive deficits [144,145]. The capacity of astrocytes to control dopamine levels depends on the expression of several proteins involved in dopamine uptake (e.g., organic cation transporters), storage (e.g., vesicular monoamine transporter 2), and metabolism (e.g., monoamine oxidase B and Catechol-O-methyltransferase). Consistently, defects in the expression and polymorphisms of these genes have been reported in patients with schizophrenia and ASD [138,146,147,148,149,150,151,152,153]. Notably, microglial cells have been found to express dopamine receptors, and a recent study has demonstrated that microglia and complement-mediated phagocytic activity play a crucial role in shaping the nucleus accumbens’ development by eliminating the D1 receptors in male, but not female rats, during adolescence. This immune-mediated elimination of the D1 receptor is necessary for natural developmental changes in social play behavior [154]. While the existence of an interplay between astrocytes and microglia based on dopamine signaling in regulating neuronal postnatal maturation has yet to be demonstrated, these data underscore the causal implication of microglia and complement-mediated immune signaling leading to developmental changes in social behavior. These findings hold significant implications for understanding the mechanisms underlying social behavior, a pivotal aspect in comprehending ASDs. Indeed, regarding microglial cells, studies have highlighted their role in inducing autism-like behaviors in mice, including repetitive behaviors and cognitive deficits [155]. Investigation into the possibility that deficits in synaptic maturation, known as ‘pruning’, may account for some of the behavioral and circuit-level deficits found in autism has revealed that reduced synaptic pruning due to the deletion of CX3CR1 on microglia during development is associated with persistent deficits in synaptic multiplicity, reduced functional connectivity between brain regions, impaired social interaction, and increased grooming behavior [155]. Additionally, a variant Ala55Thr in CX3CR1 that potentially disrupts CX3CR1 signaling has recently been found in Japanese patients, correlating with an increased risk of SZ and ASD [156]. Furthermore, TREM2, crucial for microglia-mediated synaptic refinement during early brain development, has been implicated in enhanced excitatory neurotransmission and reduced long-range functional connectivity, along with repetitive behavior and altered sociability upon its deletion in microglial cells [94]. Notably, TREM2 protein levels were also found to be negatively correlated with the severity of symptoms in individuals affected by autism. Similarly, the interleukin-1 family cytokine interleukin-33 (IL-33), produced by developing astrocytes and necessary for normal synapse numbers and neural circuit development [97], has been implicated in various neurodevelopmental and psychiatric disorders, including ASD and SZ [157].

While the etiology of ASDs and SZ, and neurodevelopmental disorders in general, undoubtedly bears a substantial genetic component [158,159,160,161], it is essential to recognize that genetics alone cannot account for the full spectrum of ASDs and SZ [158,162], though the translation of strictly genetic insights into effective therapeutic strategies has proven to be a challenging endeavor [163]. Beyond genetic factors, emerging research underscores the pivotal role of environmental influences in the pathophysiology of neurodevelopmental disorders. Various components comprising the environment encompass psychosocial, biological, and physical factors that influence an individual from the earliest stages of life, from conception and prenatal development to birth and maturation. The significance of these environmental variations becomes more apparent when considering their interaction with genetic factors. Indeed, environmental factors, when combined with genetic susceptibilities, have come under scrutiny as potential contributors to the development of conditions like ASD and SZ. While traditional views perceive genes and environmental influences as additive, the newest understandings highlight their interactive nature [159,162,164]. This updated perspective acknowledges that environmental factors can have diverse impacts, contingent on an individual’s specific genetic makeup, and vice versa. Notably, the existence of seminal papers suggesting the contributions of genetic, nutritional, immunological, and toxic environmental factors to ASD and SZ does not eliminate the social environment as having a critical role in neural development. Recognizing the intertwining roles of genetics and the environment could also shed light on the nature of certain environmental risk factors. Researchers might benefit from a comprehensive exploration of the entire “envirome” to identify the various environmental risk factors associated with ASDs and SZ, mirroring the way geneticists scrutinize the complete genome for all the genes affecting susceptibility to the disorders. This comprehensive approach considers the intricate interplay of genetic and environmental variables, including the possible impact of perinatal inflammation. By unraveling the collective influence and interactions stemming from the genome and the envirome, we can envision a comprehensive understanding of the multifaceted puzzle that is ASD and SZ. Presently, two notable features of the envirome, namely, maternal immune dysregulation factors and delivery/birth complications, are believed to hold potential as risk factors for ASD and SZ [159,162,164].

Numerous studies have suggested a potential association between viral infection during pregnancy and an elevated prevalence of ASD and SZ in offspring [13,14,165,166,167]. In rodent models of maternal immune activation (MIA), the offspring of pregnant mice exposed to viral infection or synthetic double-stranded RNA (dsRNA) poly(I:C)—an inducer of viral infection-like responses—exhibited ASD-like behavioral manifestations such as impaired social interaction, communication deficits, and repetitive behaviors [168], as well as SZ-like symptoms including deficits in working memory and behavioral flexibility [10,169]. These models have been pivotal in elucidating the molecular mechanisms influencing early neurodevelopment [10], particularly changes in the complement system, an extensively studied signaling pathway involved in the regulation of synaptic pruning by glial cells [9,170]. Such alterations contribute to dysfunctions in neuronal connectivity and synaptic pruning, which may contribute to the pathological features observed in the postmortem brains of individuals with SZ and ASD, including reduced cortical gray matter thickness and social deficits [170,171].

MIA can induce inflammatory responses in both microglia and astrocytes, leading to changes in their functional states [172,173]. Specifically, astrocytes may become hyperactivated, resulting in the release of elevated levels of proinflammatory cytokines and ATP. Microglia, equipped with P2Y and cytokine receptors, are sensitive to these alterations and can reciprocally modulate their responses [74,75]. This bidirectional communication through purinergic signaling can have significant implications for neurodevelopment. Excessive cytokine release by hyperactivated microglia may influence astrocytic responses, potentially impacting astrocyte maturation and their roles in synaptic maintenance.

## 5. Conclusions and Future Perspectives

Considering the comprehensive analysis of the relationship between microglia and astrocytes at synapses, it becomes increasingly apparent that their intricate interplay plays a pivotal role in the pathogenesis of neurodevelopmental disorders such as ASD and SZ. Our in-depth exploration has revealed that dysfunctions in both microglial and astrocytic activities during critical neurodevelopmental stages can lead to a spectrum of cognitive and behavioral aberrations, contributing to the emergence of ASD and SZ phenotypes in rodent models. Moreover, the elucidation of the roles of microglial cells and astrocytes in regulating synapse formation and pruning, coupled with the identification of specific genetic variations and altered gene expressions in patients with ASD and SZ, underscores the significance of glial cells’ involvement in the etiology of these complex disorders.

Consequently, with the integration of the evolving model systems, including human-induced pluripotent stem cell (hiPSC)-derived microglia and astrocytes, there arises a significant opportunity to unravel the dynamic interactions between these glial cells in the context of ASDs or SZ [174]. By incorporating cutting-edge methodologies such as single-cell analyses, spatial transcriptomics, multi-omics, and advanced imaging techniques, we can gain a more nuanced understanding of the underlying cellular and molecular mechanisms driving the pathology of these disorders.

Furthermore, it is evident that therapeutic strategies aimed at modulating the activities of microglia and astrocytes, along with their intricate interplay, hold significant promise for mitigating the detrimental effects of neuroinflammation and synaptic dysregulation in ASDs and SZ [175,176,177,178]. Future research efforts will likely focus on leveraging anti-inflammatory therapies, fine-tuning microglial synaptic pruning, and aiming to restore the delicate balance of the neuroimmune environment.

In conclusion, the collaborative efforts to decode the interdependent roles of microglia and astrocytes in the context of ASDs and SZ have illuminated promising avenues for therapeutic interventions, underscoring the importance of further research and translational approaches that bridge the gap between preclinical insights and clinical applications.
